# Transpiration rates of red maple (*Acer rubrum* L.) differ between management contexts in urban forests of Maryland, USA

**DOI:** 10.1038/s41598-021-01804-3

**Published:** 2021-11-18

**Authors:** Sarah Ponte, Nancy F. Sonti, Tuana H. Phillips, Mitchell A. Pavao-Zuckerman

**Affiliations:** 1 Department of Environmental Science and Technology, University of Maryland, 1428 Anim. Sci/Agr. Eng Bldg., College Park, MD 20742 USA; 2 USDA Forest Service, Northern Research Station, Baltimore, MD USA

**Keywords:** Urban ecology, Hydrology, Ecosystem ecology, Forest ecology, Urban ecology

## Abstract

The hydrological functioning of urban trees can reduce stormwater runoff, mitigate the risk of flood, and improve water quality in developed areas. Tree canopies intercept rainfall and return water to the atmosphere through transpiration, while roots increase infiltration and storage in the soil. Despite this, the amount of stormwater that trees remove through these functions in urban settings is not well characterized, limiting the use of urban forests as practical stormwater management strategies. To address this gap, we use ecohydrological approaches to assess the transpiration rates of urban trees in different management settings. Our research questions are: Do transpiration rates of trees of the same species vary among different management contexts? Do relationships between environmental drivers and transpiration change among management contexts? These management settings included single trees over turfgrass and a cluster of trees over turfgrass in Montgomery County, MD, and closed canopy forest with a leaf litter layer in Baltimore, MD. We used sap flux sensors installed in 18 mature red maple (*Acer rubrum* L.) trees to characterize transpiration rates during the growing season. We also measured soil volumetric water content, air temperature, relative humidity, and precipitation at each site. In agreement with our initial hypothesis, we found that single trees had nearly three times the daily sum of sap flux density (*J*_*S*_) of closed canopy trees. When averaged over the entire measurement period, *J*_*S*_ was approximately 260, 195, and 91 g H_2_O cm^−2^ day^−1^ for single trees, cluster trees and closed canopy trees, respectively. Additionally, single trees were more responsive to VPD than closed canopy and cluster trees. These results provide a better understanding of the influence of management context on urban tree transpiration and can help to identify targets to better manage urban forest settings to reduce urban stormwater runoff.

## Introduction

Urbanization dramatically alters hydrologic systems by replacing vegetated areas with impervious surfaces, reducing stream complexity, and altering overall water and sediment budgets^1^. Increases in impervious surfaces generate increased stormwater runoff that carries pollutants (e.g., metals, nutrients, and sediments) from built surfaces into nearby water bodies and ultimately to larger watersheds^2,3^. Consequently, impaired water quality, flooding, and eroded stream banks are major problems caused by stormwater runoff^4^. Stormwater runoff management is seen as crucial to promote greater water quality, healthier communities, and to prevent flooding in urban areas^1,5^. Mitigating urban impacts on hydrology have historically relied upon engineered solutions, but recent approaches have seen innovations in integrating ecological design and nature-based solutions into cities^1,6^.

The hydrological functions of trees and forests can reduce stormwater runoff, lessen the risk of flooding, and improve water quality in developed areas. However, urban forests (here defined following Konijnendijk et al.^7^ as all trees and woody plants located in an urban area, including street trees, woodlots, and remnant patches) have not historically been viewed as part of new urban planning solutions to environmental challenges, such as green infrastructure^8^. An exception to this includes tree boxes that are designed engineering features that act as parts of distributed green stormwater infrastructure systems^9,10^. Common green stormwater infrastructure types include green roofs, bioretention systems, rain gardens, rainwater harvesting systems, bioswales, and permeable pavement^11,12^. These strategies promote a wide variety of ecosystem services by altering hydrologic, energetic, and biogeochemical fluxes in built environments. Green stormwater infrastructure is designed to address urban water quality and quantity issues through flood control, bioretention, and groundwater recharge, but also can support secondary ecosystem services, such as carbon sequestration, climate change adaptation, biodiversity enhancement, improvement of human well-being, and increased socio-ecological connectivity^13–15^. Urban forests and trees are looked to as providers of many of these ecosystem services as well. Urban forests can manage stormwater through a host of ecohydrologic mechanisms including: evaporation, transpiration, interception of runoff, throughfall and stemflow, and stormwater storage in the soil^16^. Additionally, by transpiring water out of the soil, trees increase soil water-holding capacity for future storm events^17^. This capacity can be substantial, for example, in Baltimore, forest patch soils have the capacity to infiltrate 68% of rainfall events^18^.

Drivers of urban forest ecohydrology may differ from non-urban forests due to altered environmental conditions such as soil compaction, lack of forest understory^17^, increased evapotranspirative demand^19^, higher water vapor pressure deficit (VPD)^20^, and consequently increased evaporation and transpiration rates due to the oasis effect^21–24^. Moreover, the amount of stormwater runoff removed by urban trees through ecohydrologic mechanisms is not well established, limiting the use of trees and forests as practical stormwater management strategies. Species differences in anatomy and physiology likely influence stormwater runoff reductions due to differences in transpiration rates and response to environmental drivers^25,26^. Evapotranspiration plays an important role in the reduction of stormwater runoff and water resources conservation. The loss of vegetation through processes of urbanization is thought to contribute to stormwater and surface water management issues through the reduction of evapotranspiration^27^. However, the heterogeneity of urban landscapes and the spatial variability of evapotranspiration creates a challenge for the extrapolation of hydrological measurements of urban forests across areas with different structures^28^. Urban development may change the spatial configuration and patch size of treed areas^29^. Runoff reduction by urban trees therefore varies based on local management strategies and contexts that can influence the nature of forest canopies^17^.

Within the urban mosaic, trees exist in urban forest settings with varying structures from individual, open-grown trees to trees in relatively intact forest stands^7^. These urban forest contexts vary with respect to species composition, density, canopy cover, and arrangement^30^. Individual trees in these varying contexts differ with respect to their architecture and growth patterns, ultimately influencing the functional ecology of both trees and urban forests^31,32^. Tree water use can thus be affected by tree species and management context^33^. *Management context* refers to the variable patterns, characteristics, and distribution of trees across the urban landscape, where an urban tree can be located in a forest patch, a park, a residential yard, or along a sidewalk^7^. Management context implies that there is a specific urban environment surrounding trees in different locations that reflects ecological interactions and management decisions, and that these environments will influence tree architecture, urban forest structure, plant physiology, and ecohydrology. These management contexts therefore affect critical urban ecosystem processes and services, such as transpiration^21,34^, carbon sequestration^35^, and air pollution removal^36,37^. There is considerable variability in the quantification of ecosystem services from urban trees (such as, runoff reduction and water quality benefits) that results in part from the variation in the management context of urban trees and forests^17^. Understanding how different management contexts affect urban ecohydrologic fluxes, such as transpiration, can aid the development of policy on the application and effectiveness of urban tree canopy as a tool for stormwater runoff reduction at watershed and city scales^17^.

In this study, we aimed to determine if transpiration rates of urban trees of the same species vary among different management contexts and if the relationship between environmental drivers and transpiration changes across these contexts. Here, we use management context to mean different urban tree configurations with respect to canopy structure, stem density, ground cover, and location in the built environment that would affect sap flux density. To determine how urban management context influences transpiration rates, we instrumented red maple (*Acer rubrum* L.) trees (n = 18 trees total) with sensors to monitor sap flux density from June to November at sites (n = 3 sites) that represent different urban management contexts in Maryland. We hypothesized that transpiration rates of trees of the same species would be greater in more open contexts and be more responsive to climate drivers than those located in closed canopy contexts. Overall, we provide evidence for the importance of the role of management context on ecohydrologic fluxes of urban forests. These results highlight the importance of spatial heterogeneity of management contexts within cities for urban forestry^7^ and watershed management approaches aimed at increasing urban tree cover^8,16,17^.

## Results

### Microclimate data

**Figure 1:**
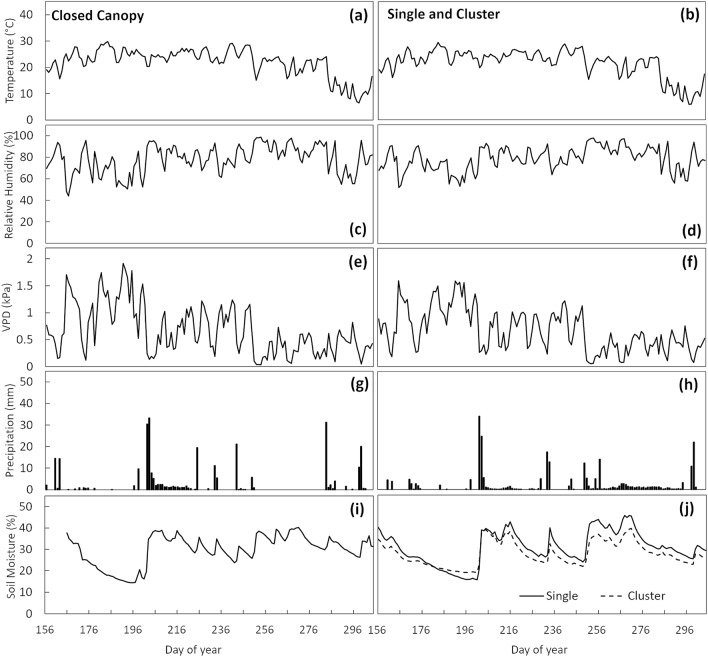
Microclimate in Baltimore, MD and Gaithersburg, MD from June to December of 2018. Mean daily air temperature for closed canopy site (**a**) and single and cluster sites (**b**); mean daily relative humidity for closed canopy site (**c**) and single and cluster sites (**d**); mean daily vapor-pressure deficit (VPD) for closed canopy site (**e**) and single and cluster sites (**f**); daily total precipitation for closed canopy site (**g**) and single and cluster sites (**h**); mean daily soil moisture in the closed canopy (**i**), single and cluster sites (**j**). Due to the later date of soil moisture sensors installation, there was a gap in the data for the closed canopy site.

We collected microclimate data from June through November, 2018. Overall, microclimatic conditions were similar across study sites. The main daily air temperature ranged from 6 to 30 °C and the average temperature over the entire period was 22 °C for the study sites (Fig. 1a,b). The average relative humidity was 78.1% for the closed canopy site and 78.4% for the single and cluster sites (Fig. 1c,d). The calculated VPD ranged from 0.03 to 1.9 kPa, with the average calculated VPD being 0.67 kPa for the study sites (Fig. 1e,f). The total amount of rainfall during the study was 284 mm for the closed canopy site and 251 mm for the single and cluster sites. The average daily soil moisture ranged from 16 to 45%, 19–39%, and 14–40% in the single, cluster and closed canopy sites, respectively (Fig. 1i,j). The average soil moisture content was 30.6, 27.7, and 29.8% in the single, cluster and closed canopy sites, respectively (Table 1).

**Table 1:** Characteristics of study trees, including tree size, canopy width north–south (N–S) and east–west (E–W), daily sum of sap flux (*J*_*S*_), and daily volumetric water content (VWC).

Management context	n	DBH (cm)	Height (m)	Canopy width N–S (m)	Canopy width E–W (m)	*J*_*S*_ (g cm^−2^ day^−1^)	VWC (%)
Single trees	5	22.4 ± 2.4	8.2 ± 0.3	8.2 ± 0.8	8.7 ± 0.7	260.4 ± 5.4	30.6 ± 0.6
Cluster of trees	4	24.7 ± 0.6	12.7 ± 0.5	9.8 ± 0.6	9.6 ± 0.5	195.3 ± 7.7	27.7 ± 0.5
Closed canopy	9	34.1 ± 4.4	21.0 ± 1.9	9.7 ± 1.0	9.0 ± 1.0	91.5 ± 2.1	29.8 ± 0.6

Values are mean ± SE.

### Sap flux density

**Figure 2:**
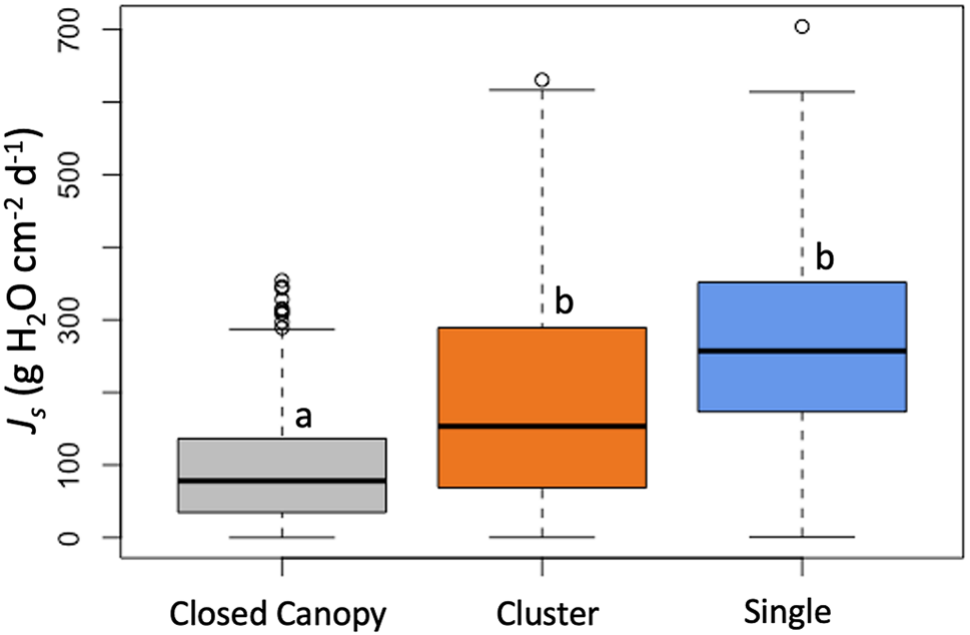
Box plots of the daily sum of sap flux density (*J*_*S*_) across the three management contexts. Unique letters above boxes indicate a significant difference among treatments based on post hoc analysis. (Figure created using R software version 4.0.5^65^).

**Figure 3:**
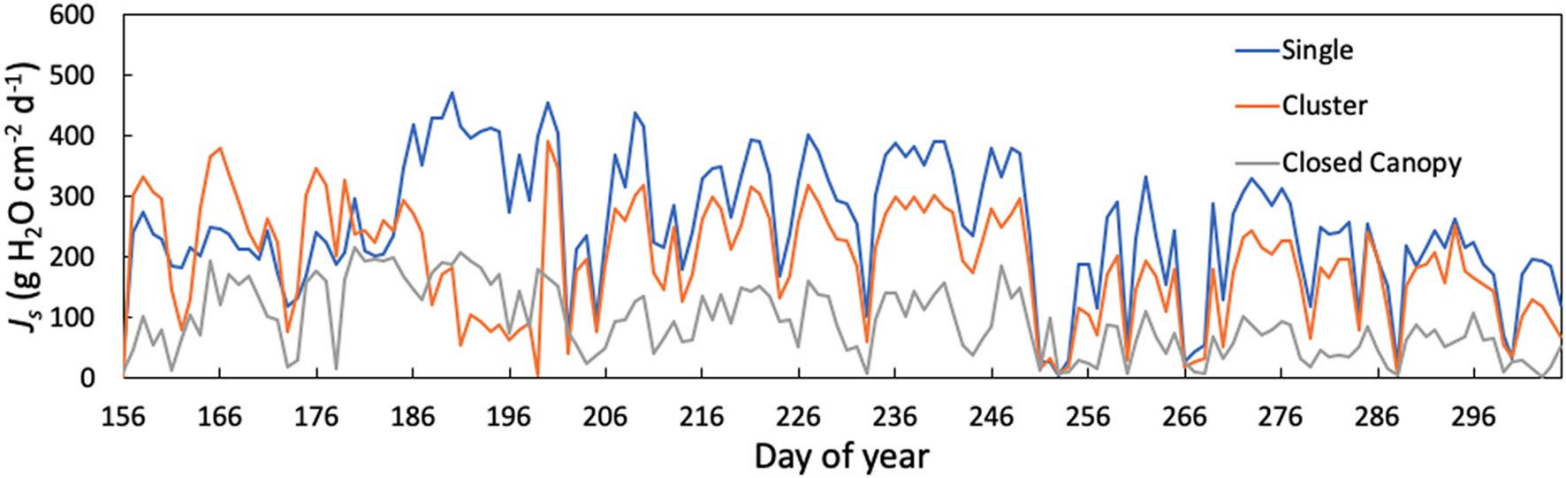
Time-series of the mean daily sum of sap flux density (*J*_*S*_) in the outer 2 cm measured in 2018 for the three management contexts.

Management context significantly impacted daily sum of sap flux density (*J*_*S;*_ Fig. 2, repeated measures ANOVA, *p* value < 0.0001). There were pairwise differences between the single and the closed canopy sites and between the cluster and the closed canopy sites. The median *J*_*S*_ for the single site was over three times the median *J*_*S*_ in the closed canopy site (257.2 compared to 78.0 g H_2_0 cm^−2^ day^−1^; Fig. 2). The single site had the highest *J*_*S*_ across the study sites (Fig. 3). The mean *J*_*S*_ at the single site was nearly three times the amount for the closed canopy site (Table 1).

### Relationships between sap flux and environmental drivers

Sap flux data were split into three 7-week time periods to investigate the seasonal variation of *J*_*S*_ in response to soil moisture and VPD (Tables 2, 3). Linear regression was used to determine the association between the independent variable (soil moisture or VPD) and dependent variable (*J*_*S*_). *J*_*S*_ was not significantly related to soil moisture, except early and late summer at the single tree and closed canopy sites (Table 3). However, based on the R^2^ values of these relationships, soil moisture explains very little of the variability of *J*_*S*_ (R^2^ values between 0.001 and 0.17, Table 3).

**Table 2:** Average parameters and R^2^ of daily sum of sap flux density (*J*_*S*_) versus daily average vapor pressure deficit (VPD) relationships of the form y = a * ln(VPD) + b.

Time period	Dates	Single			Cluster			Closed Canopy		
		a	b	R^2^	a	b	R^2^	a	b	R^2^
Early Summer	June 5–July 24	121.9 ± 13.5^a^	297.5 ± 38.2^a^	0.28 ± 0.10	94.6 ± 52.4^ab^	270.7 ± 113.6^ab^	0.21 ± 0.12	66.4 ± 7.7^b^	147.3 ± 15.5^b^	0.66 ± 0.03
Late Summer	July 25–Sept 12	139.7 ± 12.5^a^	370.1 ± 35.8^a^	0.88 ± 0.03	104.9 ± 46.3^ab^	281.9 ± 115.1^ab^	0.76 ± 0.16	40.5 ± 3.4^b^	122.1 ± 12.2^b^	0.52 ± 0.04
Fall	Sept 13–Nov 1	117.4 ± 11.5^a^	326.5 ± 27.3^a^	0.72 ± 0.03	96.9 ± 37.8^ab^	252.4 ± 92.6^ab^	0.61 ± 0.12	35.3 ± 4.6^b^	93.8 ± 12.1^b^	0.58 ± 0.05

Values are mean ± SE. Unique letters indicate cross site differences within each time period based on post hoc analysis.

**Table 3:** Regression parameters and R^2^ of daily sum of sap flux density (*J*_*S*_) vs. daily average soil moisture content relationships of the form y = a * (soil moisture) + b.

Time period	Dates	Single				Cluster				Closed canopy			
		a	b	R^2^	*p*	a	b	R^2^	*p*	a	b	R^2^	*p*
Early Summer	June 5–July 24	**− 9.7**	**509.5**	**0.17**	**< 0.001**	3.1	155.5	0.01	0.40	**− 3.9**	**225.3**	**0.14**	**< 0.001**
Late Summer	July 25–Sept 12	**− 5.6**	**476.1**	**0.07**	**< 0.001**	**− **4.7	353.6	0.02	0.07	**− 2.1**	**165.6**	**0.03**	**0.002**
Fall	Sept 13–Nov 1	0.8	169.8	0.00	0.46	**− **1.2	179.4	0.00	0.58	0.2	45.0	0.00	0.69

Significant relationships are indicated in bold font.

**Figure 4:**
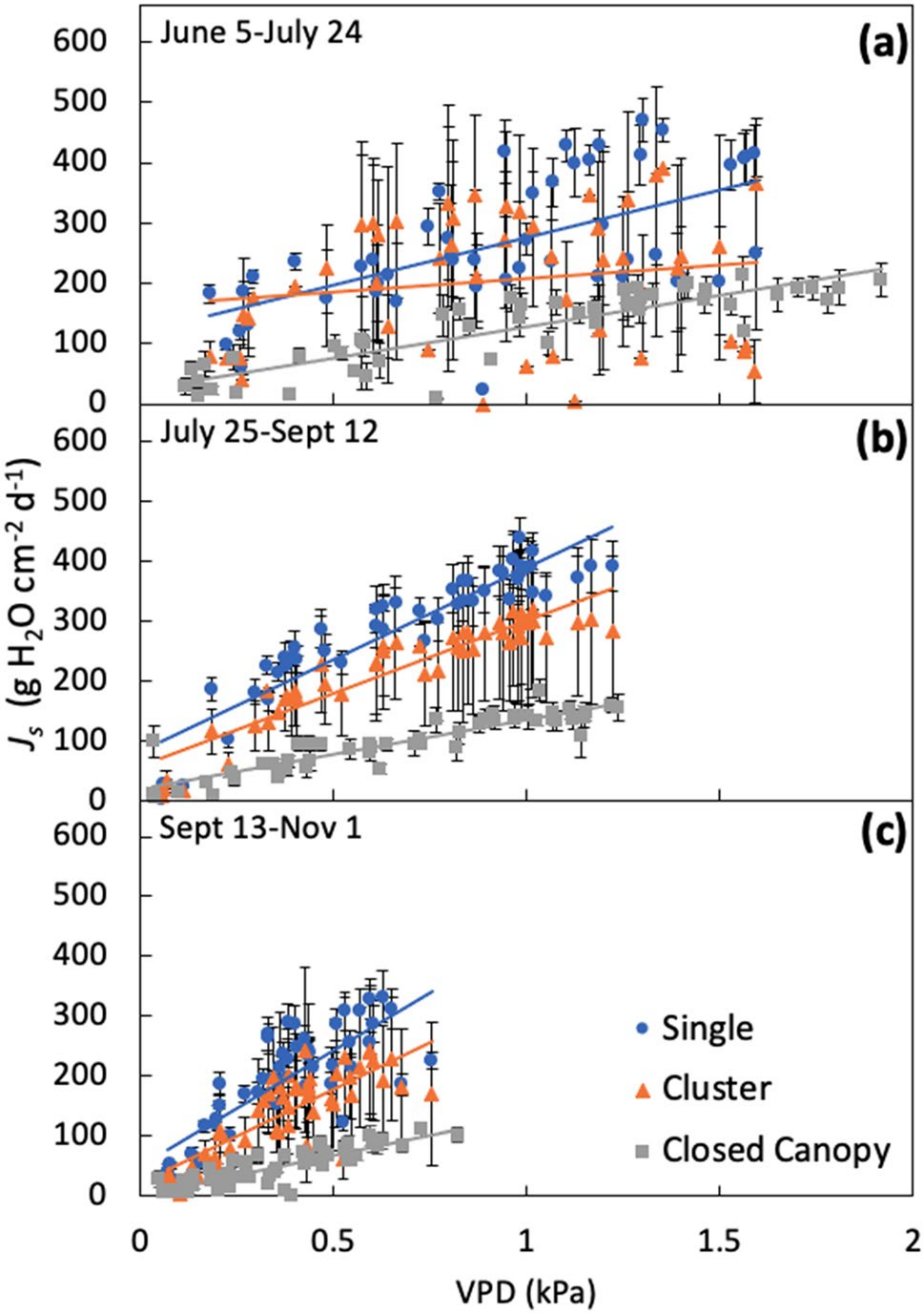
Mean daily sum of sap flux density (*J*_*S*_) as a function of VPD during early summer (**a**), late summer (**b**), and fall (**c**). Values are mean ± SE.

Repeated measures ANOVA of the *J*_*S*_-VPD regression parameters showed that the slope and intercept were significantly different between single and closed canopy sites across all time periods (Table 2, Fig. 4). The slope and intercept for the single and cluster sites were more than twice the values at the closed canopy site during the late summer and fall (Table 2). Based on the R^2^, VPD explains more of the variability of *J*_*S*_ later in the summer when compared to early summer for the single and cluster sites, but this was not observed for the closed canopy site (Table 2, Fig. 4). When comparing the regression parameters for each management context across the time periods, the slope for the closed canopy site was significantly different between early summer and late summer (*p* = 0.001), and between early summer and fall (*p* = 0.001). For the intercept, there was a significant difference between early summer and fall (*p* = 0.009) for the closed canopy site.

During the early summer, VPD showed the most variation (Fig. 4a), while the fall period had less variation (Fig. 4c). This pattern is consistent with the change in relative humidity and temperature variation over time (Fig. 1). During the late summer (Fig. 4b) and fall (Fig. 4c), single trees had a higher *J*_*S*_ for the same VPD when compared to cluster and closed canopy trees.

## Discussion

In this study, we investigated the impacts of management contexts on red maple transpiration rates and the relationship between environmental drivers and transpiration to determine the effects of different urban tree configurations on *J*_*S*_. We observed that management context significantly impacted *J*_*S*_ and that single trees over turfgrass had the highest transpiration rates, while trees at the closed canopy site had the lowest transpiration rates as estimated by sap flux monitoring results. Trees at the single and cluster sites had greater variability in *J*_*S*_ than at the closed canopy site. Additionally, there were seasonal differences between VPD and *J*_*S*_ across the management contexts.

Similar to our findings, previous urban hydrology and meteorology studies have shown that isolated trees in urban areas may have increased transpiration over more densely vegetated areas due to the oasis or leading-edge effect, where enhanced transpiration is caused by exposure to advection of strong VPD^21–24,38^. Our results are also consistent with a previous study where California sycamore street trees showed the highest sap flux density when compared to trees in irrigated and unmanaged forest sites; during the mid and late summer periods, daily sap flux density and whole-tree transpiration at the street tree site was approximately twice the amount as their natural and irrigated sites^39^. Crowding and shading from neighboring trees have significant effects on tree architecture^40^. These effects on architecture may cause differences in transpiration rates across different tree configurations and management contexts. The greater variability in *J*_*S*_ observed for the single and cluster sites (Fig. 2) might suggest that microclimate factors greatly impact urban tree transpiration rates of sites less buffered (e.g., fewer canopy layers and lower tree density) from environmental drivers.

*J*_*S*_ across management contexts was not closely related to soil moisture content (Fig. 4). Soil moisture contents also did not vary greatly between management contexts but were responsive to precipitation events between sites (Fig. 1). It is important to note that we measured soil moisture content only in surface soils to a depth of 30 cm and within the dripline of trees that we instrumented with sap flux probes. The trees in our study are mature and not grown in constructed tree pits and thus have root systems that extend meters deep into soil horizons^41^. It is likely then that the transpirational dynamics of the trees we monitored are disconnected from the water dynamics in the surface soils. Well-established mature trees, including trees in urban areas therefore are drawing water from soil depths below our measurements^42^. Indeed, irrigated trees in urban contexts are likely using ground water—Urban trees also can draw on groundwater to survive drought conditions^43^. While measurements beyond surface soil depths were beyond the scope of our study, an improved understanding of the water sources utilized by urban trees is critical for ultimately linking the dynamics of transpiration with stormwater runoff reduction.

The relationship between VPD and *J*_*S*_ differed among management contexts and this relationship also varied across time periods. VPD was a strong driver of transpiration, consistent with previous studies that reported VPD as a dominant factor affecting tree transpiration^44–48^. Urban site factors such as soil compaction, restricted rooting space, and water stress, may play a role in influencing stomatal conductance and transpiration rates^39,49^. A study in Seattle, WA reported that, due to restricted stomatal conductance, whole-tree transpiration for sweetgum trees was lower in a paved plaza site, where there was less water availability and a higher VPD when compared to park trees^50^. From our regression analyses, the single and cluster sites had a steeper slope than the closed canopy site across all time periods (Table 2). This suggests that individual trees and clusters of trees over turfgrass are more responsive to VPD than trees in forest patches. Additionally, the larger intercept for the single and cluster sites when compared to the closed canopy site across all time periods can be explained by a reduced humidity at the leaf surface. Increasing stand density promotes a decrease on the rate of transpiration of individual leaves due to shielding from the microclimate (e.g., radiation and wind)^51^, so that single and cluster sites are less buffered from environmental drivers might explain why the increase of R^2^ from early summer to late summer was not observed for the closed canopy site (Table 2).

While our study documents the relationship between *J*_*S*_ for individual trees and urban management context, stormwater management using urban forests will also require an understanding of whole-tree transpiration and forest stand structure. Wullschleger et al.^52^ reported that the whole-tree sap flow for mature red maple trees in an upland oak forest in the eastern U.S. was greatest for large trees due to the sapwood thickness. Increased radiation due to the position within the forest canopy also tended to promote higher rates of sap flow for larger trees; net radiation and atmospheric humidity deficit were the main climatic variables influencing canopy transpiration^52^. Under greenhouse conditions, red maple trees experienced reduced growth and transpiration with reduced hydraulic flow and increased soil compaction, showing the influence of soil bulk density on transpiration rates^49^. Additionally, tree transpiration may be regulated by leaf-specific root hydraulic conductance, foliar biomass, and whole plant hydraulic conductance^46^. Acknowledging this, a better understanding of runoff reduction by urban trees could integrate our context-specific findings with research that considers how trees interact with respect to shared root systems, overlapping leaf area, and branch architecture in closed versus open-grown sites.

Currently, there is limited empirical research that investigates transpiration rates of urban trees^17^. According to a modeling study, transpiration was the main process through which tree canopy contributed to runoff reduction, by removing soil water and increasing soil pore space availability for infiltration^53^. Previous work has shown that the design of bioretention suspended pavement systems influenced transpiration rates for a deciduous conifer tree (*Taxodium distichum* L.)^48^. Further long-term research using direct measurements is necessary to evaluate transpiration rates of other tree species across different urban management contexts to capture the effects of the heterogeneity and complexity of urban environments on physiological function of urban trees, and consequently, on ecohydrological processes.

This study provides insight into how different management contexts and microclimate factors influence ecohydrological fluxes of a common urban tree species. This is a crucial consideration for developing stormwater crediting programs because urban trees can be more accurately incorporated into planning efforts, increasing the effectiveness of green stormwater infrastructure networks^16^. For example, the best management practice of expanded urban tree canopy through the maintenance of existing tree canopy and/or an increase in tree cover is part of the Phase 6 Chesapeake Bay Watershed Model; therefore, a stormwater crediting method and value that takes into account the site type (e.g., an isolated tree versus a patch of trees) will better represent urban spatial heterogeneity^17^. Stormwater retention trading systems can be used as a market strategy to assign value to stormwater benefits by trees, increase tree planting efforts, and motivate further research on species selection and spacing of trees^8,54^. While single trees in our study showed the highest individual transpiration rates, urban forest patches with a greater canopy density and/or larger trees than open-grown sites will have a higher amount of transpiration per unit ground area. Understory vegetation may also contribute to the total transpiration in forest patch sites^55,56^. Thus, spatial distribution and scale of urban forests may play an important role in quantifying the influence of urban trees on stormwater management. Additionally, other ecosystem processes and services (e.g., habitat provision, biodiversity, and human well-being) occurring in highly complex urban environments may be considered in a comprehensive approach to urban forest management.

## Materials and methods

### Study sites

**Figure 5:**
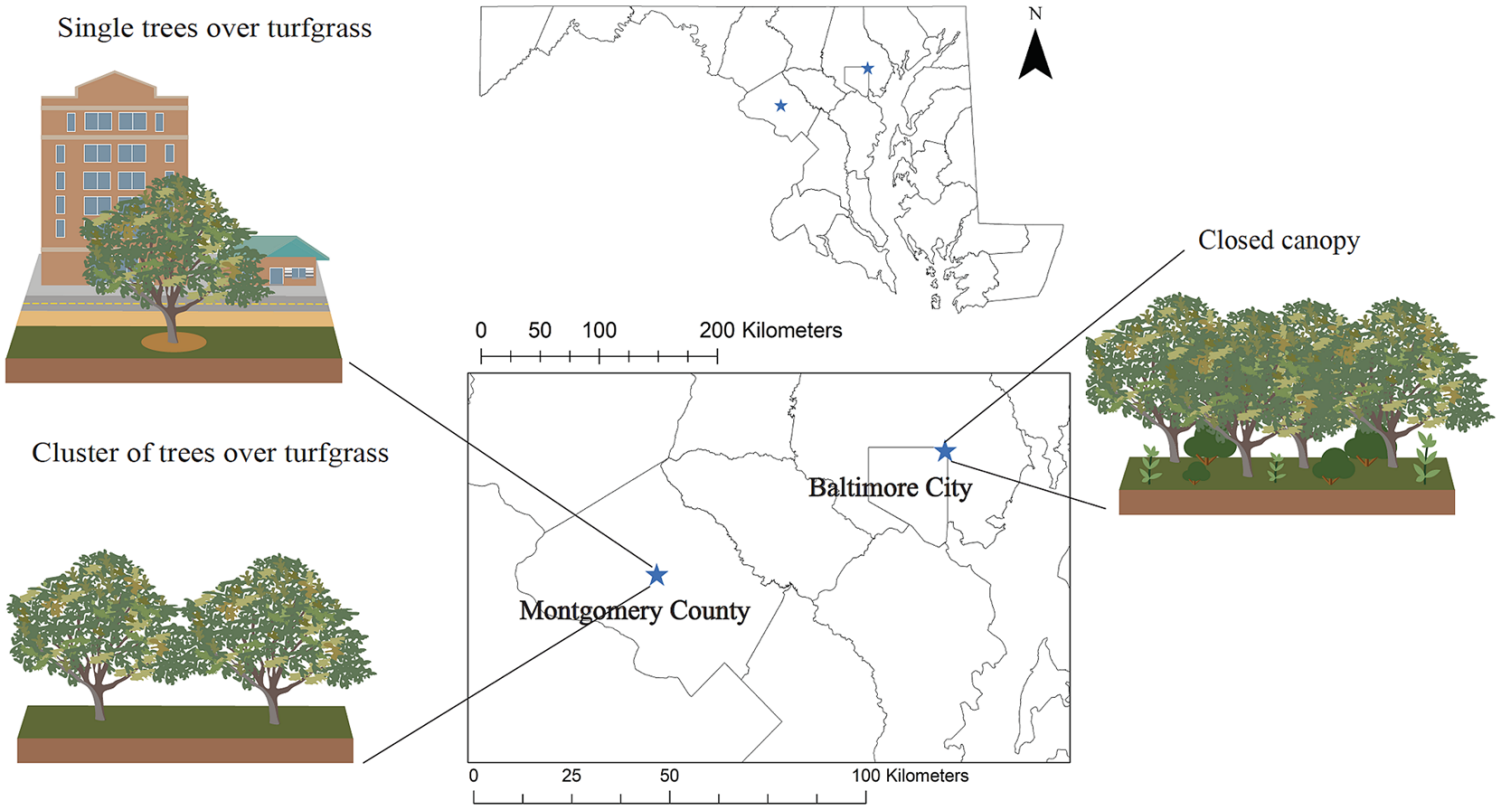
Study site locations in the State of Maryland, USA. Illustrations represent the management contexts in each site.

We conducted this study in 2018 at two cities in Maryland, USA (Baltimore and Gaithersburg) that reflect three urban forest management contexts: single trees over turfgrass, cluster of trees over turfgrass, and trees in a closed canopy forest with a leaf litter layer (Fig. 5). Data collection began in June 2018. All sites were located in the Piedmont Plateau physiographic province. In this province, soils are deep, moderately sloping, well-drained upland underlain by semi-basic, mixed basic, and acidic rocks; the dominant piedmont soils are Ultic Hapludalfs. Maximum evapotranspiration occurs in the month of July, and groundwater recharge occurs between mid-September and March^57^. Baltimore City receives an annual average of 119 cm precipitation with an annual average temperature of 13.6 °C, while Gaithersburg receives an annual average of 114.5 cm precipitation^58^.

The field sites were located at Asbury Methodist Village, a retirement community in Gaithersburg, MD (39° 08′ 59.5″ N, 77° 11′ 44.8″ W) and at the Maryland School for the Blind in Baltimore, MD (39° 21′ 59.7″ N, 76° 32′ 06.7″ W). At Asbury Methodist Village, sap flux density was measured from trees in a cluster of trees over turfgrass (n = 4) and trees over turfgrass along the sidewalk (n = 5). At the Maryland School for the Blind, sap flux density was measured from two closed canopy forest patches, which varied in size (e.g., DBH, canopy cover, and leaf area index). Sap flux density was measured from trees at the southeast side (n = 4) and at the northwest side of campus (n = 5). Red maple (*Acer rubrum* L.) was chosen as the focal species for this study because it is a commonly found tree species in urban areas throughout the eastern US. The field sites were selected based upon site accessibility and safety and the ability to provide security for the monitoring equipment. Permission to access and instrument the red maple study trees was obtained from properties owners. CITES (Convention on International Trade in Endangered Species of Wild Fauna and Flora) and IUCN policies on research involving species at risk of extinction are not applicable to the species used in this study.

### Atmospheric and soil moisture measurements

A weather station (HOBO U30 Station; Onset Computer Corporation, Bourne, MA, USA) was installed in the open (outside the forest canopy) at each field location to characterize potential climate drivers of sap flux density, including air temperature, relative humidity, and precipitation. Vapor-pressure deficit (VPD) was calculated based on air temperature and relative humidity measured at 30-min intervals^59^. In addition, point measurements of soil moisture (Campbell Scientific CS616) were installed vertically in the soil profile to a depth of 30 cm at the dripline of each tree.

### Sap flux measurements

Thermal dissipation Granier sensors^60^ were inserted into the sapwood of the 18 monitored red maple trees at a height of 1.4 m. Each sensor was constructed from two cylindrical probes of 2 mm diameter, and they were inserted 2 cm into the sapwood of the bole. The probes were placed approximately 15 cm apart. The upper probe was equipped with a constant heating wire supplied with a constant power source. Sensors were attached to double-shielded cable wires and connected to a CR 1000 data logger (Campbell Scientific Inc., Logan, UT) with AM 16/32B and AM 416 multiplexers (Campbell Scientific Inc., Logan, UT). The voltage differential (∆V) between the upper heated probe and the ambient temperature of the below reference probe was calculated every 30 s and recorded as half-hour averages. Sensors were covered with aluminum shielding to prevent interference from thermal heating or rainwater. Sap flux density in the outer 2 cm of xylem (J_*S*_, g H_2_O cm^−2^ s^−1^) was calculated according to the empirical formula^61^ modified from Granier^60,62^ (Eq. 1):

1 $$J_{S} = 119\left( {\frac{{\Delta Vmax -\Delta V}}{{\Delta V}}} \right)^{1.231}$$

To obtain daily sum of sap flux density (J_*S*_), we scaled the half-hourly measurements from the 1-s instantaneous values to a half hourly value and added all the values to get to g H_2_0 cm^−2^ day^−1^.

We used the software Baseliner 4.0^63^ to convert the data stored in the data logger to the K value and subsequently to *J*_*S*_ (Eq. 2):

2 $$K = \frac{{\Delta Vmax{ } - { }\Delta V}}{{\Delta V}}$$

where ∆V_max_ is the maximum voltage differential between probes when flux is zero, and K is a dimensionless “flow index”.

### Statistical data analysis

Repeated measures analysis of variance (ANOVA) using linear mixed-effects models^64^ was performed to compare J_*S*_ across management contexts and the effect of day of the year. Each tree was treated as a random intercept in the models and repeated measurements on the same tree over time were accounted for using autocorrelation-moving average correlation structure. Assumptions of normality and homoscedasticity were checked using QQ and residuals diagnostics plots.

Models were fit in the statistical R software version 4.0.5^65^ using the lme function in the nlme package^66^. The final model was chosen based on the smallest value of Akaike information criterion (AIC) from alternative model specifications. The varIdent function, allowing for different variances per management context, was incorporated into the model to accommodate heteroscedasticity. Additionally, an autoregressive lag 1 covariance structure was incorporated to account for temporal autocorrelation of J_*S*_. Tukey's HSD post hoc analysis with α = 0.05 using the lsmeans function from the package emmeans^67^ was conducted to identify significantly different pairs.

Relationships between J_*S*_ and VPD for each 7-week time period were fit for each tree with natural logarithm functions (y = a * ln(VPD) + b) and differences across time for the slope and intercept were tested for significance (*p* value ≤ 0.05) using repeated measures ANOVA as described above. The relationship between J_*S*_ and soil moisture content (%) were fit using a linear regression model for each 7-week time period and tested for overall significance (*p* value ≤ 0.05).

## Data Availability

All data generated during and/or analyzed during the current study will be available from the corresponding author upon reasonable request.
